# Supplementary material for: Quality over Quantity: The Association Between Daily Social Interactions and Loneliness
Source: Int J Environ Res Public Health. 2025 Sep 10;22(9):1411. doi: 10.3390/ijerph22091411 (PMC12470029; doi:10.3390/ijerph22091411)
Supplement: Supplementary file 1 [file ijerph-22-01411-s001.zip › ijerph-3805931-supplementary.pdf]

SUPPLEMENTAL MATERIALS

Table S1. Linear regression predicting loneliness

	Loneliness						
	M1	M2	M3	M4	M5	M6	M7
Prop. completed EMA	-0.694*	-0.767**	-0.756**	-0.784**	-0.917**	-0.821*	
	(0.272)	(0.270)	(0.270)	(0.290)	(0.328)	(0.311)	
Women	0.140	0.107	0.116	0.120	0.163	0.108	0.093
	(0.142)	(0.156)	(0.152)	(0.143)	(0.144)	(0.147)	(0.149)
Age	-0.019	-0.016	-0.016	-0.015	-0.017	-0.015	-0.015
	(0.011)	(0.012)	(0.011)	(0.011)	(0.011)	(0.011)	(0.013)
White	0.298*	0.272	0.271	0.279	0.250	0.247	0.244
	(0.148)	(0.155)	(0.156)	(0.152)	(0.158)	(0.144)	(0.167)
Education							
HS or GED	-0.789*	-0.836*	-0.816*	-0.929*	-0.818	-0.683*	-0.855*
	(0.389)	(0.348)	(0.357)	(0.414)	(0.440)	(0.301)	(0.386)
Some college	-0.501	-0.560	-0.547	-0.652	-0.613	-0.348	-0.561
	(0.436)	(0.389)	(0.403)	(0.452)	(0.472)	(0.356)	(0.427)
College	-0.581	-0.652	-0.633	-0.756	-0.687	-0.456	-0.619
	(0.431)	(0.377)	(0.389)	(0.438)	(0.468)	(0.342)	(0.422)
Employed	-0.122	-0.111	-0.120	-0.176	-0.191	-0.179	-0.039
	(0.173)	(0.176)	(0.180)	(0.175)	(0.173)	(0.159)	(0.176)
Functional activity limitation	0.683***	0.679***	0.681***	0.598***	0.678***	0.574***	0.632***
	(0.137)	(0.139)	(0.137)	(0.135)	(0.127)	(0.132)	(0.133)
Prop. alone	0.092						
	(0.067)						
Prop. socializing		0.070					
		(0.080)					
Mean # interaction partners			0.020				
			(0.085)				
Bridging social capital				0.146			
				(0.074)			

Bonding social capital					-0.235***		
					(0.058)		
Stressful interaction						0.261***	
						(0.056)	
Network size							-0.129*
							(0.054)
Constant	1.657	1.596	1.571*	1.738*	1.828*	1.548*	1.004
	(0.877)	(0.826)	(0.780)	(0.808)	(0.809)	(0.765)	(0.905)
N	272	272	272	261	261	261	272
R-squared	0.243	0.240	0.235	0.247	0.278	0.284	0.224

Note: All social interaction measures are standardized to enable comparison of effect sizes; Standard errors in parentheses; Prop. = Proportion; HS = High school; GED = General educational development.
